# Prevalence and Antimicrobial Susceptibility Profile of Stenotrophomonas maltophilia in a Tertiary Care Hospital in Eastern India: A Five-Year Trend Analysis

**DOI:** 10.7759/cureus.110407

**Published:** 2026-06-07

**Authors:** Amresh Pati, Swaranika Mandal, Gayatree Nayak, Dipti Pattnaik, Kumudini Panigrahi, Basanti Kumari Pathi

**Affiliations:** 1 Microbiology, Kalinga Institute of Medical Sciences, Bhubaneswar, IND

**Keywords:** antimicrobial resistance, intensive care unit, levofloxacin, minocycline, non-fermenting gram-negative bacilli, stenotrophomonas maltophilia, trimethoprim-sulfamethoxazole

## Abstract

Background and objectives: *Stenotrophomonas maltophilia *(*S. maltophilia*) is an emerging pathogen causing hospital-acquired infections, particularly in critically ill and immunocompromised patients. Limited therapeutic options are available due to its multidrug resistance (MDR) nature. Despite the availability of advanced automated diagnostic methods in Eastern India, data describing its prevalence and antibiotic susceptibility patterns are limited. The present study was conducted to evaluate the prevalence, antimicrobial susceptibility patterns, and resistance trends of *S. maltophilia *over five years in a tertiary care hospital in Eastern India.

Materials and methods: A retrospective laboratory-based observational study was conducted in the Department of Microbiology at Kalinga Institute of Medical Sciences (KIMS), a tertiary care hospital in Eastern India. Records of patients with positive culture reports for *S. maltophilia* isolates between January 2021 and December 2025 were analyzed after Institutional Ethics Committee approval (KIIT/KIMS/IEC/2616/2026). Clinically significant *S. maltophilia *isolates from various clinical specimens of hospitalized patients aged >18 years were included in the study.

Samples were processed using standard microbiological procedures. Isolate identification was performed by VITEK 2 compact automated system (bioMérieux, Marcy-l'Étoile, France) and/or MALDI-TOF MS (VITEK MS PRIME system -bioMérieux, Marcy-l'Étoile, France). Antimicrobial susceptibility testing for trimethoprim-sulfamethoxazole, levofloxacin, and minocycline was performed by automated systems and interpreted according to CLSI M100, 34th edition (2024). Demographic, clinical, and laboratory data were retrieved from the laboratory information system and analyzed using R software Version 4.4.3 (R Foundation for Statistical Computing, Vienna, Austria). Categorical data were described with frequencies and percentages. A p-value of <0.05 was considered statistically significant.

Results: During the five-year period, a total of 415 clinically significant *S. maltophilia *isolates were identified from 88,315 culture-positive samples, with an overall prevalence of *S. maltophilia* infection of 0.5%. The annual prevalence of isolates increased considerably from 38/10,239 (0.37%) in 2021 to 131/20,529 (0.6%) in 2025. The majority of the isolates were from ICUs (272/415; 65.5%), and males were 281/415 (67.7%), with a median age of 54 years. Blood was the most common specimen source (138/415; 33.3%), followed by pus/tissue/wound swabs (113/415; 27.2%). During the five years, minocycline had the highest susceptibility (73.7-83.2%), followed by trimethoprim-sulfamethoxazole (66.7-74.0 %) and levofloxacin (60.5-71.4%). Most isolates remained susceptible, while resistance phenotypes persisted, especially to levofloxacin and trimethoprim-sulfamethoxazole.

Conclusions: The rising isolation rate of *S. maltophilia *from clinical specimens, particularly from bloodstream infections, is an emerging epidemiological and clinical concern. There was an observed stable but notable resistance pattern to the antibiotics, such as fluoroquinolones and trimethoprim-sulfamethoxazole, highlighting the need for continuous surveillance and prudent antibiotic use, while minocycline remained the most effective antimicrobial during the study period.

## Introduction

*Stenotrophomonas maltophilia* (*S. maltophilia*) is an aerobic, non-fermentative gram-negative bacillus that has been widely recognized as a major nosocomial pathogen within the previous three decades [1]. The organism was first identified in 1943 as *Bacterium bookeri* and was reclassified several times until it was named *Stenotrophomonas maltophilia *in 1993 [2]. The phrase is taken from the Greek roots meaning “the narrow feeder that loves malt” [3].

*S. maltophilia* is widely disseminated in the hospital environment, especially in the water system, respiratory devices, and medical equipment [4]. The organism has numerous chromosomally encoded mechanisms of intrinsic resistance to multiple antimicrobial drugs, such as multidrug efflux pumps (SmeDEF and SmeABC), β-lactamases (L1 and L2), aminoglycoside-modifying enzymes, and quinolone resistance genes [5,6]. This high resistance profile severely limits possible treatment alternatives.

The incidence of *S. maltophilia* infections has been increasing worldwide, including in India [7]. A recent multicentric Indian study analyzing 271 bloodstream infection isolates from 54 healthcare centres from 2017 to 2024 demonstrated a steady rise in the isolation rates per year, with the highest number of isolates reported in 2023-2024 (76 isolates, 28.04%) as compared to 27-38 isolates (9.96-14.02%) in the earlier years [8]. Similar growing trends have been seen in Europe, Asia, and Latin America. The factors that contributed to this increase were related to prolonged hospital stay, mechanical ventilation, central venous catheterization, and post-COVID-19 sequelae [9,10,11]. Most of the clinical isolates of *S. maltophilia* infections in Indian tertiary care hospitals have been reported from intensive care units (ICUs) and respiratory care wards [8,9].

Common clinical presentations due to *S. maltophilia* infections are pneumonia, bloodstream, urinary tract, wound, and device-associated infections [12]. Studies from South India have also documented considerable mortality linked to *S. maltophilia* bacteremia, with improvement due to the early initiation of targeted antibiotic therapy [13]. Trimethoprim-sulfamethoxazole (TMP-SMX) has been widely regarded as the first-line medication for treatment of *S. maltophilia* infections, with previous susceptibility rates greater than 95% [14]. However, growing resistance to TMP-SMX, levofloxacin, and ceftazidime has been described in the latest surveillance studies [15,16]. This increasing resistance further complicates care due to the limited number of effective therapeutic antimicrobial drugs. Alternative therapeutic antimicrobials include minocycline, tigecycline, cefiderocol, and ceftazidime-avibactam, either alone or in combination with TMP-SMX [17].

Although there is availability of improved diagnostic services, such as Vitek-2 and MALDI-TOF MS system, data on the prevalence, antibiotic susceptibility patterns, and temporal trends of *S. maltophilia* infections in Eastern India are scanty. Understanding regional epidemiology and resistance patterns is vital for the development of evidence-based empirical treatment guidelines and establishing antimicrobial stewardship initiatives. Hence, the present study was undertaken to determine the prevalence, antimicrobial susceptibility patterns, and resistance trends of *S. maltophilia* during a five-year period in a tertiary care hospital in Eastern India.

## Materials and methods

This was a retrospective, laboratory-based observational study undertaken in the Department of Microbiology at Kalinga Institute of Medical Sciences (KIMS), a tertiary care hospital in Eastern India, after getting approval from the Institutional Ethics Committee (KIIT/KIMS/IEC/2616/2026). Culture and antimicrobial susceptibility records of hospitalized patients with *S. maltophilia* isolates between January 2021 and December 2025 were analyzed. Relevant clinical details were retrieved from medical records. All specimens had been collected and processed previously in the microbiology laboratory as part of routine diagnostic procedures, and only the corresponding laboratory data were reviewed and analyzed for the study.

Study population

Clinically significant isolates of *S. maltophilia *obtained from infected adult patients (age >18 years) of either sex and isolates from specimens (blood, wound/pus, body fluids, and others, including urine, catheter tips, respiratory samples like sputum, tracheal aspirate, and bronchoalveolar lavage), and the first isolate were included. Duplicate isolates from the same patient, polymicrobial growth, environmental samples, non-sterile sites without indicators of infection, and records with missing clinical data were excluded from the study. Colonization versus infection was distinguished by definition: Colonization was defined as the isolation of *S. maltophilia* without concomitant clinical signs and symptoms of infection. Infection was defined as a positive culture with clinical evidence, such as fever, hypotension, worsening respiratory status, purulent drainage, local erythema or swelling, leukocytosis or leukopenia, elevated inflammatory markers, or radiologic evidence of infection. Supportive findings for respiratory specimens were new or increasing infiltrates, increased oxygen need, and purulent tracheal or bronchial secretions. For blood or sterile-site isolates, clinical sepsis or site-specific symptoms were used [18,19].

Study procedure

The specimens, after receiving in the Microbiology Laboratory, were processed as per standard protocols, with inoculation on appropriate media and incubation at 35-37 °C for 18-48 hours. Suspected non-fermenting Gram-negative bacilli were evaluated by Gram stain, oxidase, and a panel of biochemical reactions or subjected directly to automated identification. Definitive identification of *S. maltophilia *was performed using Vitek-2 GN identification cards and, where available, MALDI-TOF MS (VITEK MS PRIME system- bioMérieux, Marcy-l'Étoile, France). Antimicrobial susceptibility testing was performed using Vitek-2 GN N406 AST cards (bioMérieux, Marcy-l'Étoile, France), interpreted (susceptible, intermediate, and resistant) according to the recommendations of the Clinical and Laboratory Standards Institute (CLSI) M100, 34th edition (2024) guidelines [20]. The agents consistently evaluated and analyzed in this study were TMP-SMX, levofloxacin, and minocycline. Preference was given to drugs having validated breakpoints and availability in an Indian setup instead of others, such as cefiderocol [20]. *Escherichia coli* ATCC 25922 and *Pseudomonas aeruginosa* ATCC 27853 were used as the quality control strain. Quality controls of all the bacteriological AST procedures were carried out on a weekly cycle.

Data collection and statistical analysis

Data regarding patient demographics (age and sex) and microbiological culture and susceptibility results (organism and AST profile) were retrieved from electronic medical records and entered into Microsoft Excel (Microsoft Corporation, Washington, USA). Statistical analysis was performed using R software, version 4.4.3 (R Foundation for Statistical Computing, Vienna, Austria). Categorical variables were presented as frequencies and percentages, and the prevalence of *S. maltophilia* was compared using the Chi-square test; p < 0.05 was considered statistically significant.

## Results

During the stipulated time period of five years (January 2021 to December 2025), 415 clinically relevant *S. maltophilia* isolates were identified from 88,315 culture-positive samples, giving an overall prevalence of 0.5%. The annual prevalence of isolates increased considerably from 38/10,239 (0.37%) in 2021 to 131/20,529 (0.6%) in 2025.

Table 1 shows the distribution of *S. maltophilia* isolates among the study population (n = 415). The median age of the study participants was 54 years, and the ICU patients were significantly older (58.2 years) than the non-ICU patients (48.6 years) (p < 0.001). Male patients were predominantly infected (67.7% of cases; p < 0.001). The majority of the isolates were obtained from the ICU (272/415; 65.5%), a rising trend in the isolation proportion was recorded from 2021 to 2025, in both the ICU and non-ICU groups. The proportion of *S. maltophilia* isolates increased progressively over the years, from 38/415 (9.2%) in 2021 to 131/415 (31.6%) in 2025, showing a statistically significant upward trend (p < 0.001). Among the clinical specimens, blood was the most common clinical specimen source, accounting for 138 (33.3%) isolates, followed by pus/tissue/wound swabs (113; 27.2%). The specimen type distribution of the isolates was statistically significant (p < 0.001).

**Table 1:** Distribution of Stenotrophomonas maltophilia among the study population Descriptive variables were expressed as frequency and percentage (N%). The Chi-square test was used, and Chi-square values were given along with the p values. A p-value of <0.05 was considered statistically significant.

Parameter	Total (n = 415)	ICU (n = 272)	Non-ICU (n = 143)	Statistical test used	Test statistics	p-value
Age (in years)	54.0 ± 9.5	58.2 ± 8.7	48.6 ± 11.8	Unpaired t-test	8.329	< 0.001
Gender						
Male	281 (67.7%)	194 (71.3%)	87 (60.8%)	Chi-square test	17.694	< 0.001
Female	134 (32.3%)	78 (28.7%)	56 (39.2%)			
Year						
2021	38 (9.2%)	23 (8.5%)	15 (10.5%)	Chi-square test	21.036	< 0.001
2022	49 (11.8%)	31 (11.4%)	18 (12.6%)			
2023	78 (18.8%)	53 (19.5%)	25 (17.5%)			
2024	119 (28.7%)	78 (28.7%)	41 (28.7%)			
2025	131 (31.6%)	87 (32.0%)	44 (30.8%)			
Specimen						
Blood	138 (33.3%)	95 (34.9%)	43 (30.1%)	Chi-square test	19.427	< 0.001
Body fluid	56 (13.5%)	38 (14.0%)	18 (12.6%)			
Pus, tissue, and wound swabs	113 (27.2%)	81 (29.8%)	32 (22.4%)			
Others	108 (26.0%)	58 (21.3%)	50 (35.0%)			

Table 2 shows the findings of the antimicrobial susceptibility profile of* S. maltophilia *isolates against levofloxacin (LVX), minocycline (MI), and SXT over the five-year study period (n = 415).

**Table 2:** AST findings of Stenotrphomonas maltophilia (n = 415) AST: antimicrobial susceptibility test; LVX: levofloxacin; MI: minocycline; SXT: cotrimoxazole

Year	LVX	MI	SXT
Year 2021 (n = 38)			
Susceptible	23 (60.5%)	28 (73.7%)	26 (68.4%)
Intermediate	6 (15.8%)	7 (18.4%)	5 (13.2%)
Resistant	9 (23.7%)	3 (7.9%)	7 (18.4%)
Year 2022 (n = 49)			
Susceptible	35 (71.4%)	39 (79.6%)	34 (69.4%)
Intermediate	8 (16.3%)	6 (12.2%)	7 (14.3%)
Resistant	6 (12.2%)	4 (8.2%)	8 (16.3%)
Year 2023 (n = 78)			
Susceptible	54 (69.2%)	61 (78.2%)	52 (66.7%)
Intermediate	14 (17.9%)	11 (14.1%)	19 (24.4%)
Resistant	10 (12.8%)	6 (7.7%)	7 (8.9%)
Year 2024 (n = 119)			
Susceptible	84 (70.6%)	96 (80.7%)	86 (72.3%)
Intermediate	21 (17.6%)	14 (11.8%)	18 (15.1%)
Resistant	14 (11.8%)	9 (7.6%)	15 (12.6%)
Year 2025 (n = 131)			
Susceptible	91 (69.5%)	109 (83.2%)	97 (74.0%)
Intermediate	19 (14.5%)	9 (6.9%)	16 (12.2%)
Resistant	21 (16.0%)	13 (9.9%)	18 (13.7%)

Among all the antimicrobials tested against *S. maltophilia*, higher susceptibility rates were seen in minocycline (28-109; 73.7-83.2%), followed by SXT (26-97; 66.7-74.0%) and levofloxacin (23-91; 60.5-71.4%). Minocycline resistance was low throughout the study period, while fluoroquinolone (e.g., levofloxacin) resistance rates were relatively greater, particularly in the beginning years of the investigation. Although the number of isolates increased during 2024 and 2025, the overall susceptibility profiles among the antimicrobials evaluated for *S. maltophilia* remained reasonably stable throughout, especially for minocycline and SXT.

Table 3 shows the AST findings of *S. maltophilia* among the ICU patients. Among the isolates of ICU patients, year-wise antimicrobial susceptibility analysis showed that minocycline (MI) remained the most effective antibiotic throughout the study period, with susceptibility rates ranging from 19/23 (82.6%) in 2021 to 76/87 (87.4%) in 2025. SXT also showed good and sustained activity, with susceptibility ranging from 18/23 (78.3%) in 2021 to 68/87 (78.2%) in 2025. LVX showed comparatively lower susceptibility than other antimicrobials, ranging from 14/23 (60.9%) in 2021 to 63/87 (72.4%) in 2025.

**Table 3:** AST findings of Stenotrophomonas maltophilia among the ICU patients (n = 272) AST: antimicrobial susceptibility test; LVX: levofloxacin; MI: minocycline; SXT: cotrimoxazole

Year	LVX	MI	SXT
Year 2021 (n = 23)			
Susceptible	14 (60.9%)	19 (82.6%)	18 (78.3%)
Intermediate	2 (8.7%)	2 (8.7%)	1 (4.3%)
Resistant	7 (30.4%)	2 (8.7%)	4 (17.4%)
Year 2022 (n = 31)			
Susceptible	24 (77.4%)	29 (93.5%)	25 (80.6%)
Intermediate	2 (6.5%)	1 (3.2%)	1 (3.2%)
Resistant	5 (16.1%)	1 (3.2%)	5 (16.1%)
Year 2023 (n = 53)			
Susceptible	36 (67.9%)	44 (83.0%)	37 (69.8%)
Intermediate	9 (17.0%)	5 (9.4%)	12 (22.6%)
Resistant	8 (15.1%)	4 (7.5%)	4 (7.5%)
Year 2024 (n = 78)			
Susceptible	54 (69.2%)	63 (80.8%)	55 (70.5%)
Intermediate	14 (17.9%)	9 (11.5%)	12 (15.4%)
Resistant	10 (12.8%)	6 (7.7%)	11 (14.1%)
Year 2025 (n = 87)			
Susceptible	63 (72.4%)	76 (87.4%)	68 (78.2%)
Intermediate	8 (9.2%)	2 (2.3%)	7 (8.0%)
Resistant	16 (18.4%)	9 (10.3%)	12 (13.8%)

Table 4 shows the AST findings of *S. maltophilia* among non-ICU patients. Year-wise antimicrobial susceptibility analysis showed that susceptibility to LVX increased from 9/15 (60.0%) in 2021 to 30/41 (73.2%) in 2024, followed by a slight decline to 28/44 (63.6%) in 2025. MI showed comparatively higher susceptibility throughout the study period, ranging from 9/15 (60.0%) in 2021 to 33/41 (80.5%) in 2024 and 33/44 (75.0%) in 2025. SXT showed moderate susceptibility, varying from 8/15 (53.3%) in 2021 to 31/41 (75.6%) in 2024.

**Table 4:** AST findings of Stenotrophomonas maltophilia among the non-ICU patients (n = 143) AST: antimicrobial susceptibility test; LVX: levofloxacin; MI: minocycline; SXT: cotrimoxazole

Year	LVX	MI	SXT
Year 2021 (n = 15)			
Susceptible	9 (60.0%)	9 (60.0%)	8 (53.3%)
Intermediate	4 (26.7%)	5 (33.3%)	4 (26.7%)
Resistant	2 (13.3%)	1 (6.7%)	3 (20.0%)
Year 2022 (n = 18)			
Susceptible	11 (61.1%)	10 (55.6%)	9 (50.0%)
Intermediate	6 (33.3%)	5 (27.8%)	6 (33.3%)
Resistant	1 (5.6%)	3 (16.7%)	3 (16.7%)
Year 2023 (n = 25)			
Susceptible	18 (72.0%)	17 (68.0%)	15 (60.0%)
Intermediate	5 (20.0%)	6 (24.0%)	7 (28%)
Resistant	2 (8.0%)	2 (8.0%)	3 (12%)
Year 2024 (n = 41)			
Susceptible	30 (73.2%)	33 (80.5%)	31 (75.6%)
Intermediate	7 (17.1%)	5 (12.2%)	6 (14.6%)
Resistant	4 (9.8%)	3 (7.3%)	4 (9.8%)
Year 2025 (n = 44)			
Susceptible	28 (63.6%)	33 (75.0%)	29 (65.9%)
Intermediate	11 (25.0%)	7 (15.9%)	9 (20.5%)
Resistant	5 (11.4%)	4 (9.1%)	6 (13.6%)

## Discussion

The present study demonstrates a progressive rise in *S. maltophilia *isolates from different infections over the five-year period, with prevalence rising from 38/10,239 (0.37%) in 2021 to 131/20,529 (0.6%) in 2025, and has emerged as an increasingly common nosocomial pathogen in a tertiary care hospital of Eastern India.

Similar increasing trends have been reported in Indian and global studies, highlighting the growing importance of *S. maltophilia* as a nosocomial pathogen [8,19,21]. The increased isolation rate may be attributed to prolonged hospitalization, increased ICU admissions, extensive use of broad-spectrum antibiotics, invasive procedures, and improved diagnostic facilities [13,18,22].

Most of the isolates (65.5%) were recovered from ICU patients, and infected patients from ICUs were older than those from non-ICUs (58.2 vs. 48.6 years; p < 0.001). These findings support the known association of *S. maltophilia* with critically ill patients commonly exposed to invasive devices and prolonged antimicrobial therapy [18,19]. Isolation was common among male patients in the present study, which is consistent with previous reports [8,21]. The increasing isolation of the organism outside ICU settings in this study also suggests its emergence as a clinically significant pathogen beyond critically ill patients [18,19]. Moreover, the bacteria are described as an emerging pathogen of public health concern [9].

Bloodstream infections constituted a major proportion of isolates (33.3%), followed by pus/tissue/wound specimens (27.2%). The predominance of bloodstream isolates highlights the invasive potential of the organism [18,19]. The increasing isolation trend in both ICU and non-ICU settings further suggests its emergence beyond critical care units [9]. Similar distribution patterns were also reported in the studies done in India by Singh et al. [8] and Jacob et al. [13].

A considerable fraction of wounds and tissues also indicates their involvement in post-operative or device-related wound infections. This adaptability is well known in *S. maltophilia* as a versatile environmental organism that has the ability to contaminate hospital water, disinfectants, and equipment and cause infection in vulnerable hosts.

Susceptibility toward minocycline was found to be highest throughout the study period, ranging from 73.7% in 2021 to 83.2% in 2025, followed by TMP-SMX. Levofloxacin demonstrated comparatively lower susceptibility and higher resistance rates, particularly during the earlier years of the study. Similar patterns of susceptibility have been reported by different studies, reflecting increasing fluoroquinolone resistance among *S. maltophilia* isolates [14,21,23,24]. Among ICU isolates, minocycline and TMP-SMX retained good activity throughout the study period, whereas levofloxacin showed comparatively lower susceptibility. Similar trends were observed among non-ICU isolates. These findings support the continued role of minocycline and TMP-SMX as effective therapeutic options for *S. maltophilia* infections.

Despite increasing isolation rates, the antimicrobial susceptibility profiles remained relatively stable during the study period. This may be indicative of effective infection control practices and judicious use of antimicrobials. Also, these findings underscore the need for standardized empiric therapy guidelines across hospital settings with TMP-SMX as first-line therapy and minocycline reserved for severe or resistant infections.

Strengths and limitations

The strengths of this study include its five-year span, and the relatively large number of isolates provides a robust overview of local epidemiology with the antimicrobial susceptibility profile of *S. maltophilia* in a tertiary care hospital. The main limitations of this study include its retrospective design, reliance on laboratory data without systematic clinical correlation of infection versus colonization, and the absence of detailed outcome and antibiotic-exposure information. Since this study was carried out in one center only, it also lacks data on the molecular mechanisms of resistance. Further multicenter investigations integrating genomic and phenotypic analyses are strongly recommended to better understand the epidemiology and resistance dynamics of this emerging pathogen.

## Conclusions

*S. maltophilia *has emerged as an important nosocomial pathogen in this tertiary care hospital in Eastern India, with a rising trend of isolation over the five-year study period, particularly among ICU patients and invasive infections. Most isolates remained susceptible to TMP-SMX and minocycline, supporting their continued role as effective treatment options, while lower susceptibility to levofloxacin warrants cautious use. Continuous surveillance, institution-specific antibiograms, antimicrobial stewardship, and strict infection control practices are essential to limit the spread of *S. maltophilia *and preserve therapeutic efficacy.
